# Supplementary material for: Parietal-specific activation reveals pain from inadequate levels of altitude acclimatization/ adaptation
Source: PLoS One. 2025 Jul 15;20(7):e0326385. doi: 10.1371/journal.pone.0326385 (PMC12262904; doi:10.1371/journal.pone.0326385)
Supplement: Attachment — Submitted filename: renamed_26f4d.pdf [file pone.0326385.s001.pdf]

西藏大学医学伦理审查委员会
生物医学研究项目伦理审批件

伦研批第 (ZDYXLL2024003) 号

<table>
<tr><td>项目名称</td><td colspan="5">短期驻留人员高原适应性评估研究及应用</td></tr>
<tr><td>项目类别</td><td colspan="5">基础医学☑临床医学口预防医学口护理口药物口其他口</td></tr>
<tr><td>项目来源</td><td colspan="3">申报项目</td><td>经 费</td><td>待定</td></tr>
<tr><td>申办单位</td><td colspan="3">西藏大学</td><td>主要负责人</td><td>李昊</td></tr>
<tr><td>研究部门</td><td>教育学院</td><td>项目负责人</td><td>李昊</td><td>职 称</td><td>讲师</td></tr>
<tr><td colspan="6">伦理审查意见</td></tr>
<tr><td colspan="4">▲同意</td><td colspan="2">▲</td></tr>
<tr><td colspan="4">▲修改后同意</td><td colspan="2"></td></tr>
<tr><td colspan="4">▲不同意(项目终止或暂停)</td><td colspan="2"></td></tr>
</table>

审批意见

依据《中华人民共和国民法典》《中华人民共和国执业医师法》《医疗机构管理条例》《中华人民共和国基木医疗卫生与健康促进法》《中华人民共和囚个人信息保护法》《中华人民共和国药品管理法》《中华人民共和国数据安全法》(中华人民共和国人类遗传资源管理条例》《药物临床试验质量管理规范》《沙及人体的生物医学研究伦理审查办法》《药物临床试验伦理审查工作指导原则》《医疗卫生机构开展研究者发起的临床研究管理办法》(试行)、世界医学会《林尔辛基宜》世界卫生组织《生物医学研究审查伦理委员会操作指南》国际医学科学组织委员会《沙及人的生物医学研究国际伦理准则》等法律行政法规、部门规章、规范性文件和国际伦理准则，全体参公委员同意该项目的伦理审核申请，经木伦理委员会审查，同意按研究方案开展木项研究。

请道循伦理委员会批准的方案开展研究，保护受试者的健康与权益。

1. 研究获批后，研究过程中若变更项目负责人，对研究方案、知情同意书、调查问卷招募材料等的任何修改，请提交修正案审查中请；

2. 请按照相关法律法规规定以及研究方案中对于安全性事件报告计划，及时向西减大学医学伦班委员会提交书面不良事件报告 ；

3. 研究者没有遵从方案开展研究，可能对受试者的权益健康、以及研究的科学性造成不良影[illegible]，[illegible]提交业规事作报告；

4. 申请人暂停或提前终止研究，请及时提交暂停中止研究报告；

5. 研究如果沙及(中华人民共和国人类遗传资源管理条例》中的相关活动，请务[illegible]遵照[illegible]例要求开[illegible]相关[illegible]究

6. 研究获批后，根据相关规定中请伦理审查，木批件仅对当前的中请负责。

西藏大学医学伦理审查委员会
科研处

主任委员(签名) [illegible]

20[illegible]年1月1日

扫描全能王创建

注意：

1.“同意”的研究应遵循已经伦理委员会批准的方案执行，应符合SFDA/GCP和《赫尔辛基宣言》的原则。

2.“修改后同意”的研究方案在提交复审方案前，应按评审意见进行逐条修改，并在修改处做出标记或说明，修改后的方案连同初审意见一并递交伦理委员会申请复审。

3.“不同意”或“暂停或终止”的研究方案，申办者和研究者可就伦理委员会的意见和建议中提及的问题做出书面申诉并陈述理由。伦理委员会可就申诉做重新审查。

4.研究过程中，对研究方案和知情同意书等相关文件所做的任何修改均需得到伦理委员会审查同意后方可实施。

5.发生的严重不良事件或意外事件需向SFDA上报的同时向伦理委员会做出书面通报，伦理委员会有权根据对其评估做出新的决定。

扫描全能王创建

共

西藏大学医学伦理审查委员会

伦理评审会议情况

会议时间：2024年4月28日下午

会议地点：腾讯会议

委员会成员	性别	职务	专业	工作单位	签名	投票情况
熊海	男	常务副院长	神经病学	西藏大学医学院		委员数： 15 人<br>出席委员数：9 人<br>同意：9 票<br>修改后同意：0 票<br>不同意 ： 0票<br>弃权：0票
格桑顿珠	男	院长	神经外科	西藏自治区第二人民医院		
平措达吉	男	处长	历史	西藏大学科研处		
边巴卓玛	女	副院长	妇产科	拉萨市人民医院		
扎西达娃	男	点长	卫生事业管理	西藏大学医学院		
边巴次仁	男	所长	药学	西藏藏医院		
鲜恩英	女	无	妇产科	西藏大学医学院		
袁瑞瑛	女	无	药学	西藏大学医学院		
高飞	男	科长	计算机	西藏大学科研处		

扫描全能王创建
