# Supplementary material for: Parietal-specific activation reveals pain from inadequate levels of altitude acclimatization/ adaptation
Source: PLoS One. 2025 Jul 15;20(7):e0326385. doi: 10.1371/journal.pone.0326385 (PMC12262904; doi:10.1371/journal.pone.0326385)
Supplement: Attachment — Submitted filename: PONE-D-24-39022_review.pdf [file pone.0326385.s002.pdf]

The study explores the relationship between pain perception and acclimatization to high altitudes (HA). Researchers recruited 65 young adults living in Tibet to assess physiological measures (oxygen saturation and hematocrit levels) and brain activity via EEG. Participants were classified into high and low altitude acclimatization index (AAI) groups. Those with higher AAI reported lower pain levels, suggesting better acclimatization reduces pain perception at HA. EEG analysis revealed that parietal lobe brainwaves in delta, theta, and beta frequencies mediate the AAI-pain relationship, with increased frequency power correlating with reduced pain. This highlights the parietal cortex's role in sensory processing and pain adaptation, offering insights into non-pathological pain management strategies for those living or traveling at HA.

Page 1

~~« low-pressure~~ cold »

~~« significant temperature fluctuations »~~

Page 3

« Alpha waves »

Page 4

« Brain oscillations in the five bands »

« The central sensistization observed during low ~~altitude~~ HA ~~acclimation~~ acclimatization »

« mediating role in the relationship between altitude ~~acclimation~~ acclimatization »

« resulting from low levels of altitude ~~adaption~~ adaptation »

« Through the assessment of altitude ~~adaption~~ adaptation levels »

« a lower altitude ~~adaption~~ adaptation level is correlated with increased pain sensations »

« in the relationship between altitude ~~adaption~~ adaptation and plateau pain »

« This helps to identify the potential correlation between altitude ~~adaption~~ adaptation and pain »

Page 5

2.1 Participants

What altitude is Tibet university ? How long had participants lived at this altitude ? You seem to use the words acclimatization and adaptation as synonyms while they are not. Adaptation refers to a processus taking place over several generations. Did you include participants originating from high altitude areas ?

Did you control for pain medication or other medication that could interfere with pain perception or with altitude acclimatization ?

2.2 Recording equipment

You should specify the number of EEG electrodes (32 I guess).

Why did you chose an eye closed condition over eyes open or both conditions ?

What time of the day did you record EEG ?

2.3 Data pre-processing

You should specify the duration of time periods.

Subsequently, ~~the~~ data ~~was~~ were segmented into time peridos, with ~~the~~ contamined time periods manually labeled and excluded from further ~~analysis~~ analyses.

« rs-EEG data contamined by various sources of **physiological**, including muscle **contraction and** cardiac **signal**, and **environmental** noise were processed »

Page 6

2.4 Analysis of EEG
The analysis involved examining the power of each channel within specific frequency bands, including delta (**δ ;** 1-3Hz)), theta (**θ ;** 4-7Hz), alpha (**α ;** 8-13Hz), beta (**β ;** 14-30Hz) and gamma (**γ ;** 31-80Hz).

2.5 Pain sensation measurement

You should specify « pain **perception** or **perceived** pain »

« ~~The~~ **This** self-rating »

2.6 Altitude acclimatization measurement

« reflecting higher SpO2 and**/or** lower HCT »

Page 7

Before the results section, you should had a paragraph describing the statistical analyses that were performed : main outcome, type of test(s), significance level, power, sample size, etc.

You should had a table detailing :

- age (mean, SD, median, lowest, highest),
- duration of altitude exposure (mean, SD median, lowest, highest)
- AAI score (mean, SD, median, lowest, highest)

- P-FIBS score (mean, SD median, lowest, highest)
- EEG power in each band and total EEG power (mean, SD median, lowest, highest)

3.1 Comparison of differences in pain sensation at the AAI

« indicating that the low-AAI group perceived pain ~~frequency~~ more intensely than the high-AAI group. »

You performed the analysis on the top 30 and the bottom 30, what happened to the 5 other participants ? According to the table 1, there is no significant correlation between AAI and P-FIBS score. Then, why did you perform regression analyses were conducted to examine the mediating effects of AAI on pain ?

3.3 The mediating effect of parietal EEG between AAI and pain

What do you mean by « marginally significant » ? If your sample size is high enough to reach the suitable statistical power, then if p-value is beyond the cut-off (usually $p < 0.05$), the result is not significant.

« positive association with δ (β=0.25, p=0.04), ~~θ (β=0.23, p=0.07),~~ and β (β=0.26, p=0.04) » (p=0.07 is not significant)

Page 8

« The direct impact of ~~acculturation~~ **acclimatization** level on pain »

« Consequently, parietal δ, θ, and β frequencies were identified as complete mediators in the **association between AAI and pain** » : unless I am mistaken, your results do not show a clear association between AAI and pain perception.

« Prior research has indicated that **gamma band oscillations induced by** short-duration pain tends to localize in central regions »

Page 10

Discussion

Have you considered including a low altitude control group ?

« This suggest a potential **origin** of short-duration pain in the primary sensorimotor cortex » : your results show that low HA acclimatization is associated with changes in parietal cortex activity. What reasoning leads you to think that parietal cortex is the source of pain. The changes in parietal cortex activity may as well reflect the processing of pain perception.

I do not understand the last sentence : « Future research may benefit from the utilization of detection methods grounded in multimodal machine learning approaches, which could offer a more objective means of quantifying pain and facilitating differentiated analysis. » What detection methods have you in mind and how do you intend to use them in practice ?

Tables 2 and 3

Do these data come from the whole sample (n=65) or are there any missed data ?

Figure 2

What are the numbers and the numbers between parentheses ?
