# Supplementary material for: Parietal-specific activation reveals pain from inadequate levels of altitude acclimatization/ adaptation
Source: PLoS One. 2025 Jul 15;20(7):e0326385. doi: 10.1371/journal.pone.0326385 (PMC12262904; doi:10.1371/journal.pone.0326385)
Supplement: Attachment — Submitted filename: Response to Reviewers.doc [file pone.0326385.s003.doc]

**Reviewer 1**

**Page 5**

2.1 Participants

What altitude is Tibet university ? How long had participants lived at this altitude ? You seem to use the words acclimatization and adaptation as synonyms while they are not. Adaptation refers to a processus taking place over several generations. Did you include participants originating from high altitude areas ?

Response: We thank the reviewer for the kind consideration and constructive comments on our manuscript. We have carefully revised the manuscript and provided the point-by-point response below in blue. The changes in the revised manuscript have been highlighted. We hope these changes will strengthen our manuscript. Tibet University is located in Lhasa, Tibet Autonomous Region, at 3,680 meters. The participants included in this study lived continuously at this altitude for at least one month. Both adaptation and domestication were used because participants included migrants who had learned to move from lower altitudes to higher altitudes, as well as people who had lived at higher altitudes for generations.

Did you control for pain medication or other medication that could interfere with pain perception or with altitude acclimatization ?

Response: Thank you for your reminding. I did not make a detailed report on this part. I'm sorry for my negligence. Stringent control measures were implemented during the recruitment of participants to exclude those using medications that could potentially impact the results. This encompassed not only painkillers but also other pharmaceuticals that might interfere with pain perception or the process of altitude acclimation.

2.2 Recording equipment

You should specify the number of EEG electrodes (32 I guess).

Response: Thanks for your comments. In the present experimental study, the utilized equipment was the Compumedics NeuroScan SynAmps^2^ 8050, which was equipped with 64 electrodes designed specifically for electroencephalogram (EEG) data acquisition.

Why did you chose an eye closed condition over eyes open or both conditions ?

Response: Thanks for your comments. In this study, EEG data under the eyes-closed resting state were selected for collection instead of that under the eyes-open state. The main basis lies in the fact that previous studies have shown that the EEG data collected when the eyes are closed have higher validity. When in the eyes-open state, due to the input of a large amount of visual information, the activities of alpha waves (8 - 13 Hz) in the frontal lobe, parietal lobe and occipital lobe regions are significantly weakened, and their amplitudes are decreased as well. This may have an adverse impact on the accuracy and validity of EEG data, and further interfere with the reliability and scientific nature of the research results.（References：Li L and Zhang JX: Study of the Alpha Wave Differences Between Eyes-Closed and Eyes-Open Resting States. Journal of University of Electronic Science and Technology of China. 2010, (03), 450-453.

What time of the day did you record EEG ?

Response: Thanks for your comments. All the EEG data were acquired during the period from 9:00 to 11:00 am. At this time interval, the participants exhibited a state of high energy and reduced drowsiness. Concurrently, the brain was in an optimal working condition, thereby enhancing its responsiveness to the experimental tasks. This, in turn, contributed to the collection of more precise EEG signals.

2.3 Data pre-processing

You should specify the duration of time periods.

Response: Thank you for your careful review and valuable suggestions on this article. With regard to your question "the duration of the time period should be specified", we will elaborate here.

In our data analysis, we did not actually intercept or specify a specific period of time, because we performed spectral analysis of the entire 4 minutes of resting EEG data. That is, each subject's EEG data was recorded for a continuous four-minute period, and our analysis covered the entire period.

We do not set a specific time window, because we are concerned with the spectral characteristics over the entire recording period, rather than the changes over a specific time period. This setup allows us to capture the average spectral power over the entire resting state, giving us a more complete picture of the electrical activity of the subject's brain.

Thank you again for your review and suggestions. We hope that the above explanation clarifies the issue regarding the duration of the time period.

**Page 6**

2.5 Pain sensation measurement

You should specify « pain perception or perceived pain »

Response: Thank you for your reminder. In light of the evaluation content of the scale and in combination with the objective of this study, through careful analysis, it has been determined that what is involved here pertains to the assessment of pain perception. I have carried out supplementary corrections in the corresponding part to ensure the precision of the academic expression.

**Page 7**

Before the results section, you should had a paragraph describing the statistical analyses that were performed : main outcome, type of test(s), significance level, power, sample size, etc.

Response: Dear reviewer, thank you very much for finding interest in our findings and pointing out the flaws in the analyses. We have addressed your concerns in a point-by-point manner below, and hope that you will find the added information suitable and sufficient for publication.I added a paragraph before the results section to describe the statistical analysis that was carried out. The supplementary content is as follows: Data analysis was conducted using SPSS 21.0. Based on the core issue of this study which focuses on exploring the intrinsic link between the HA adaptation level and pain, the AAI index was selected as the key physiological indicator to reflect the high-altitude adaptation level, and the score of the pain frequency, intensity, and burden scale (P - FIBS) was used to quantify the pain level of individuals. The independent-samples t-test was adopted to evaluate the pain levels of individuals under different adaptation levels. Pearson correlation analysis was adopted to measure the correlation degree between the AAI and the P - FIBS score through the correlation coefficient r. *P* < 0.05 indicates that the results are significant and possess statistical significance.

Based on the theoretical frameworks of previous studies and this current study, in view of the potential crucial role of the electroencephalogram activities in different frequency bands of the parietal lobe in the conduction pathway between the AAI index and pain perception, it was selected as the mediating variable. The regression-based mediation effect analysis method was applied to construct the following models: Model 1, with the AAI index as the independent variable and pain as the dependent variable, to test the total effect; Model 2, with the AAI index as the independent variable and the electroencephalogram activities in different frequency bands of the parietal lobe as the dependent variable, to examine the influence of the independent variable on the mediating variable; Model 3, incorporating both the AAI index and the electroencephalogram activities in different frequency bands of the parietal lobe simultaneously, with pain as the dependent variable, to analyze the direct effect of the independent variable on the dependent variable and the indirect effect of the mediating variable after the introduction of the mediating variable. To obtain more robust estimates of the mediating effect and confidence intervals, the Bootstrap method was adopted, with the number of resampling times set to 5000. Through this extensive resampling process, an empirical sampling distribution of the mediating effect was constructed, and then its 95% confidence interval was determined. If this interval does not include 0, it indicates that the mediating effect is significantly present.

You should had a table detailing:

- age (mean, SD, median, lowest, highest),

- duration of altitude exposure (mean, SD median, lowest, highest)

- AAI score (mean, SD, median, lowest, highest)

- P-FIBS score (mean, SD median, lowest, highest)

- EEG power in each band and total EEG power (mean, SD median, lowest, highest)

Response: Thank you for the comments and suggestions on the manuscript. We have already made supplements in the corresponding parts of the article (**Table 1**).

**Table 1** The basic characteristic information of participants (*N*=65)

|  | Age | Duration | AAI | Delta | Theta | Alpha | Beta | Gamma | Total | P-FIBS |
| --- | --- | --- | --- | --- | --- | --- | --- | --- | --- | --- |
| *M* ± *SD* | 22.22±2.43 | 3.43±2.23 | 1.94±0.37 | 3.43±2.36 | -1.58±2.60 | 1.63 ±4.22 | -6.68±2.82 | -13.77±3.35 | -16.97±13.35 | 1.29±0.49 |
| Median | 22.00 | 3.00 | 1.91 | 3.45 | -1.81 | 1.81 | -6.99 | -14.56 | -18.85 | 1.00 |
| Lowest | 18.00 | 0.00 | 2.00 | -2.00 | -6.00 | -7.00 | -13.00 | -20.00 | -40.00 | 1.00 |
| Highest | 31.00 | 12.00 | 4.00 | 9.00 | 5.00 | 10.00 | 0.00 | -3.00 | 17.00 | 3.00 |

3.1 Comparison of differences in pain sensation at the AAI

You performed the analysis on the top 30 and the bottom 30, what happened to the 5 other participants ? According to the table 1, there is no significant correlation between AAI and P-FIBS score. Then, why did you perform regression analyses were conducted to examine the mediating effects of AAI on pain ?

Response: Thank you for the comments on the manuscript. We have carefully revised the manuscript and provided the point-by-point response below. Previously, 30 participants from both the front and back were selected for inclusion in the analysis, mainly because the adaptation indices of the five participants in the middle were exactly the same. In order to balance the number of participants in the high and low adaptation groups, the data of these five individuals were discarded. After your reminder, to ensure the integrity and accuracy of the data, the data of these five participants have now been reincorporated into the analysis. As can be seen from Table 1, there was no significant correlation between the AAI and P-FIBS scores. After introducing gender as a covariate and conducting a partial correlation analysis, a significant correlation was found between the AAI and P-FIBS scores (*p* = 0.042), and it satisfied the significance level of *p* < 0.05.

3.3 The mediating effect of parietal EEG between AAI and pain

What do you mean by «marginally significant» ? If your sample size is high enough to reach the suitable statistical power, then if p-value is beyond the cut-off (usually p < 0.05), the result is not significant.

«positive association with δ (β=0.25, p=0.04), θ (β=0.23, p=0.07), and β (β=0.26, p=0.04)» (p=0.07 is not significant)

Response: Thank you for pointing out the errors in our paper. As you precisely pointed out, in the scenario where the sample size is sufficiently large, the p-value ought to be able to attain an appropriate level of statistical power. The interpretation of effect sizes greater than 0.05 should be approached with circumspection. After incorporating gender as a covariate, θ (β = -0.59, *p* = 0.26)， the moderating effect not significant.

**Page 8**

«Consequently, parietal δ, θ, and β frequencies were identified as complete mediators in the association between AAI and pain» : unless I am mistaken, your results do not show a clear association between AAI and pain perception.

Response: Dear reviewer, thank you very much for pointing out the flaws in the presentation. We have already replied to your question carefully below. After introducing gender as a covariate and conducting a partial correlation analysis, a significant correlation was found between the AAI and P-FIBS scores (*p* = 0.04), and it satisfied the significance level of *p* < 0.05.

**Page 10**

Discussion

Have you considered including a low altitude control group ?

Response: We would like to thank you for your professional review work, constructive comments, and valuable suggestions on our manuscript. As you are concerned, this is an important issue need to be addressed, which are replied to in detail as below. This study did not set up a control group, mainly because of the significant differences in the environment, which made it difficult to effectively control the extraneous variables. As our understanding of the relationship between high-altitude adaptation and pain perception continues to expand and deepen, we will carefully consider adding a control group in subsequent studies, aiming to obtain more accurate and reliable experimental results, thereby promoting the in-depth development of research in this field and providing more valuable theoretical bases and practical guidance for high-altitude medicine and related fields.

«This suggest a potential origin of short-duration pain in the primary sensorimotor cortex» : your results show that low HA acclimatization is associated with changes in parietal cortex activity. What reasoning leads you to think that parietal cortex is the source of pain. The changes in parietal cortex activity may as well reflect the processing of pain perception.

Response: Thank you for pointing out this error. My limited understanding has led to imprecise expression. The primary somatosensory cortex is the region in the cerebral cortex responsible for receiving and processing sensory information from various parts of the body, located in the postcentral gyrus of the parietal lobe. The primary somatosensory cortex is a crucial area within the parietal lobe, handling sensory signals from the thalamus, including sensations such as pressure, temperature, tactile discrimination, and pain from all body parts. Therefore, we hypothesized that the adaptation level and pain perception in a high-altitude environment are both related to the parietal cortex. Our research findings have also corroborated this. Some brainwaves in the parietal cortex play an intermediary role in high-altitude adaptation and pain perception. Consequently, the statement "The changes in the activity of the parietal cortex may reflect the process of pain perception" is more accurate, and I have made corresponding modifications accordingly.

I do not understand the last sentence: «Future research may benefit from the utilization of detection methods grounded in multimodal machine learning approaches, which could offer a more objective means of quantifying pain and facilitating differentiated analysis. » What detection methods have you in mind and how do you intend to use them in practice ?

Response: Thank you for the comments and suggestions on the manuscript. For future research endeavors, the integration of EEG with electrocardiogram (ECG) signals and galvanic skin response (GSR) holds promise. ECG is capable of manifesting the responses of the autonomic nervous system. For instance, heart rate variability (HRV) may decrease in response to pain stimuli due to sympathetic nervous system excitation. Machine learning algorithms, such as support vector machines and convolutional neural networks (CNN) in the realm of deep learning, can be employed to extract and classify the features of these signals, thereby detecting pain states. Regarding GSR, which serves as an indicator reflecting emotional and autonomic nervous system arousal levels and is closely associated with the emotional responses induced by pain, algorithms like recurrent neural networks (RNN) can be utilized to analyze its time-series characteristics and construct a pain detection model.

**Tables 2 and 3**

Do these data come from the whole sample (n=65) or are there any missed data ?

Response: Thank you for pointing this out. I'm terribly sorry that I didn't provide a detailed explanation due to my negligence. I have carefully made the following corrections: We recruited 70 right-handed participants from September 15, 2023 to October 15, 2024, comprising Tibetans who have lived in high-altitude areas for generations and Han people who migrated to high-altitude regions. They had been studying or living in Lhasa (at an altitude of 3680 m) continuously for over one month. Five participants were excluded from the EEG analysis. Among them, three were excluded because excessive movements and muscle artifacts contaminated more than 50% of the trials, and two were excluded due to incomplete physiological data. Eventually, 65 participants (31 F, 34 M, age=22.22±2.43) were included in the analysis.

**Figure 2**

What are the numbers and the numbers between parentheses ?

Response: Thanks for your comments. The numbers outside the parentheses represent the strength of the relationships between variables. For example, in a simple mediation model, the independent variable X influences the dependent variable Y through the mediating variable M. The path coefficient from X to M indicates the degree of direct influence of X on M, and the path coefficient from M to Y reflects the intensity of the direct effect of M on Y. The product of these two path coefficients is the sum of the indirect effects. The path coefficient directly from X to Y reflects the direct effect of X on Y (the direct association that exists while considering the mediating variable). The magnitudes and signs of these coefficients illustrate whether the associations between variables are positive or negative and the closeness of the associations. For instance, a path coefficient of 0.5 indicates that for every one-unit change in the independent variable, the mediating variable (or the dependent variable) will change by 0.5 units accordingly; if it is -0.3, it represents a reverse association, meaning that when the independent variable increases by one unit, the mediating variable (or the dependent variable) will decrease by 0.3 units. The numbers inside the parentheses are the total effects, which are the sum of the direct effects and the indirect effects.

**Reviewer 2**

**Abstract:**

1.) The abstract is still too imprecise in some places. For example, it leaves open the question of why the fast beta waves associated with attention are rated the same as the delta and theta waves, although the delta and theta waves are associated with relaxation, or half-sleep and deep sleep.

Response: We would like to thank you for your professional review work, constructive comments, and valuable suggestions on our manuscript. Your time and efforts are greatly appreciated. We have strived to improve the manuscript accordingly as listed in details below. The enhancement of EEG activity might be regarded as a compensatory mechanism. It endeavors to maintain the normal operation of the body's physiological functions by increasing the efficiency of neural activities, thereby helping individuals to improve their adaptability to the high-altitude environment. The parietal lobe, as a key brain region responsible for processing sensory information throughout the body, exhibits the most significant activation state compared with other brain regions when an individual is in a high-altitude environment and the adaptation level shows dynamic changes. Notably, once the beta waves, which are highly associated with attention, are activated, the increase in an individual's alertness often indicates that the individual will experience a more intense and higher level of pain sensation. However, the activation of delta waves, which usually occur during the stage of deep sleep and can prompt the brain to maintain a relaxed state, generally means that the individual will perceive a relatively lower level of pain. Consequently, the rs-EEG power in the delta and beta frequency bands of the parietal lobe region serves as an important mediating factor in the connection between AAI and pain perception.

2.) It remains unclear in what sense the term "mediators" is used here.

Response: Thanks for your comments on the manuscript. A mediating variable is a variable that lies between the independent variable and the dependent variable. It can explain how the independent variable affects the dependent variable and plays a transmitting role in the causal chain. Briefly, if the independent variable X affects the dependent variable Y through the variable M, then M is the mediating variable. For example, in this study, in the relationship of "high-altitude adaptation level (X) → delta and beta brainwaves in the parietal lobe (M) → pain perception (Y)", the delta and beta brainwaves in the parietal lobe are the mediating variables. The influence of the independent variable does not directly act on the dependent variable but is transmitted through this intermediate link (the mediating variable). The activity changes of the delta and beta brainwaves in the parietal lobe play a mediating role between the HA adaptation level and the high-altitude pain perception. That is, the high-altitude adaptation level first causes the activity changes of the delta and beta in the parietal lobe, and this change further affects the individual's perception of pain. Mediating effect analysis can help us decompose the total effect. The total effect is the sum of the direct effect of the independent variable on the dependent variable and the indirect effect through the mediating variable. By studying the mediating variable, we can understand the proportion of the indirect effect (the part of the effect generated through the mediating variable) in the total effect in the entire causal process, thus gaining a deeper understanding of the relationship mechanism between variables.

3.) “parietal β (β = 0.26, p = 0.04), δ (β = 0.25, p = 0.04) and θ (β = 0.23, p = 0.07) powers acted as full mediators “: The p-value for Theta power is not significant (p = 0.07). Why do you state it as full mediator in this context? Either the presentation of p-values is confusing here or you should change the meaning of your sentence and take into account that Theta power is not significant.

Response: Thank you for pointing out this. Based on the guidance and suggestions provided by you, this study has reorganized and recalculated the data. During this process, it was found that the factor of gender has a significant impact on the research results. Therefore, the gender factor was incorporated into the analysis as a covariate for effective control. The results showed that θ (β = -0.26, *p* = 0.26), and this result did not reach the significance level standard. Hence, it will not be included in the effective mediating variables for analysis and discussion.

4.) Please specify "pain" in more detail. What pain locations were recorded? Is it diffuse pain or all types of pain?

Response: Thank you for pointing out this. Pain occurs frequently in the high-altitude and hypoxic environment. However, the locations where pain occurs usually lack clear directivity. Therefore, in this study, only the frequency, intensity and burden level of pain were tested, covering all pains that occurred in the high-altitude environment, while the specific locations where pain occurred were not recorded. In the future, with the continuous deepening of research on high-altitude pain, it is expected to conduct specific studies on pain in different locations.

**Introduction:**

1.) Please sum up your hypotheses only at the end of the Introduction section and not in between. In some places the introduction reads more like a collection of ideas than a finished text section. (e.g. page 1: “Therefore, we hypothesize that maladaptive structural alterations in central nervous system (CNS) cells resulting from exposure to plateau hypoxia,”; page 3: “Proposed H1: lower levels of altitude acclimatization are associated with heightened pain sensations.”; And so on)

Response: We would like to thank you for your professional review work on our manuscript. I have made corresponding modifications in accordance with your suggestions and reorganized the introduction, making this part more concise and organized. The hypotheses are placed at the end of the introduction and proposed together.

2.) The introduction is too long and detailed. The introduction would suit better to a narrative review than to an original article. Please shorten the introduction and focus on the most important facts to lead up to your hypotheses.

Response: Thank you for pointing out this. In accordance with your professional recommendations, I have conducted a comprehensive reorganization of the introduction section and implemented requisite reductions in content, thereby rendering this part more succinct and methodically structured.

3.) Why did you only record the EEG for the parietal lobe? Pain processing is usually associated primarily with the cortical areas of the somatosensory cortex, the insular cortex and the ACC. Also the frontal cortex region was found to show changes in pain-related EEG patterns (see Review below). Please explain your decision a little more clearly and name the usual recording locations for pain in the EEG. The following review provides a good overview: <https://www-frontiersin-org.translate.goog/journals/neuroscience/articles/10.3389/fnins.2023.1186418/full?_x_tr_sl=en&_x_tr_tl=de&_x_tr_hl=de&_x_tr_pto=rq>

Response: Thank you for recommending this review study. I have read it carefully and it has been of great inspiration to my research. This study conducted comprehensive observations on multiple brain lobes, including the frontal lobe, temporal lobe, occipital lobe, and parietal lobe. In contrast to the findings of the current review study, in the research on high-altitude residents, the frontal cortical region did not detect the various electroencephalogram pattern changes related to pain as described in the review, and the connectivity of theta and gamma frequencies did not show an increasing trend either. Instead, specific changes were detected in the parietal cortex. This finding indicates that individuals living in the hypoxic environment at high altitudes exhibit a high degree of dependence on the parietal lobe for processing pain sensations. Given that the pain mechanism in the hypoxic environment at high altitudes may be different from that in the plain environment, this study precisely fills this gap in the field and provides a new perspective for in-depth understanding of the pain mechanism in the high-altitude environment.

4.) In your introduction, you report about many studies assessing for pain perception during acute hypoxia exposure and also for processes during acclimatization periods of several weeks. In your study, however, you are working with a sample of high altitude residents. Please also address this group of people in the introduction. Is there evidence of an increased perception of pain in this population too? Why did you choose this sample and not choose unacclimatized people who were exposed to acute hypoxic conditions and observe them over several weeks?

Response: Thank you for the comments and suggestions on the manuscript. We have carefully revised the manuscript and provided the point-by-point response below. The participants incorporated into this study were all individuals who had continuously inhabited high-altitude regions for a minimum period of one month. I have effected the modification in the introduction section, uniformly altering it to "high-altitude residents". Currently, the majority of studies concentrate on populations acutely exposed to high altitudes, while relatively few investigations have been conducted on the non-pathological pain perception of residents with long-term high-altitude exposure. This is because the pain intensity under long-term high-altitude exposure is generally not as severe as that under acute high-altitude exposure, and its frequency is also relatively low, making it prone to being overlooked by researchers. In fact, such pain exerts a significant impact on the learning and daily lives of highland residents. The underlying mechanisms of pain occurrence are complex and difficult to monitor. Distinguishing between non-pathological and pathological pain is of great significance for maintaining the overall well-being of high-altitude populations. Therefore, this study focuses on the relationship between different adaptation levels and pain perception among residents who have been living in high-altitude areas for a long time.

**Methods:**

1.) When reading the method section it becomes clear that you not only examined the parietal lobe, but also other cortex areas using EEG. Please make this fact clear earlier (e.g. in the abstract)! Same for Alpha and Gamma waves.

Response: Thanks for your comments. According to your professional suggestions, I have made the following changes: the resting-state electroencephalogram (rs-EEG) signals of all frequency bands including delta, theta, alpha, beta and gamma were acquired from the frontal lobe, parietal lobe, temporal lobe and occipital lobe.

2.) The description of your statistical analyses is completely missing in the Methods section. Please add a corresponding text section!

Response: We would like to thank you for your professional review work, constructive comments, and valuable suggestions on our manuscript. Your time and efforts are greatly appreciated. We have strived to improve the manuscript accordingly as listed in details below. I have added Section 2.7 Statistical Analysis to the methodology part: Data analysis was conducted using SPSS 21.0. Based on the core issue of this study which focuses on exploring the intrinsic link between the HA adaptation level and pain, the AAI index was selected as the key physiological indicator to reflect the high-altitude adaptation level, and the score of the pain frequency, intensity, and burden scale (P - FIBS) was used to quantify the pain level of individuals. The independent-samples *t*-test was adopted to evaluate the pain levels of individuals under different adaptation levels. Pearson correlation analysis was adopted to measure the correlation degree between the AAI and the P - FIBS score through the correlation coefficient (r). *P* < 0.05 indicates that the results are significant and possess statistical significance.

Based on the theoretical frameworks of previous studies and this current study, in view of the potential crucial role of the electroencephalogram activities in different frequency bands of the parietal lobe in the conduction pathway between the AAI index and pain perception, it was selected as the mediating variable. The regression-based mediation effect analysis method was applied to construct the following models: Model 1, with the AAI index as the independent variable and pain as the dependent variable, to test the total effect; Model 2, with the AAI index as the independent variable and the electroencephalogram activities in different frequency bands of the parietal lobe as the dependent variable, to examine the influence of the independent variable on the mediating variable; Model 3, incorporating both the AAI index and the electroencephalogram activities in different frequency bands of the parietal lobe simultaneously, with pain as the dependent variable, to analyze the direct effect of the independent variable on the dependent variable and the indirect effect of the mediating variable after the introduction of the mediating variable. To obtain more robust estimates of the mediating effect and confidence intervals, the Bootstrap method was adopted, with the number of resampling times set to 5000. Through this extensive resampling process, an empirical sampling distribution of the mediating effect was constructed, and then its 95% confidence interval was determined. If this interval does not include 0, it indicates that the mediating effect is significantly present.

**Results:**

1.) Page 8: “The direct impact of acculturation level on pain, in the presence of mediating factors, was determined to be statistically insignificant (p>0.1).”: Did you use a significance level of 0.1 for all statistical calculations? Please specify your approach in a separate text section in the Methods section. Also explain why you did not choose the mostly accepted significance level of 0.05!

Response: Thank you for pointing out the errors in our paper. As you precisely pointed out, in the scenario where the sample size is sufficiently large, the *p*-value ought to be able to attain an appropriate level of statistical power. The interpretation of effect sizes greater than 0.05 should be approached with circumspection. After incorporating gender as a covariate, the moderating effect not significant, θ (β=-0.59, *p*=0.26). In addition, the most commonly accepted significance level of 0.05 was uniformly adopted throughout the whole text.

2.) Figure 2: Are you showing p-values or r-values here? Or both?

Response: Thanks for your comments. In order to ensure the neatness and aesthetics of the path diagram, the P-values and r-values of the pairwise relationships among the independent variables, mediating variables, and dependent variables were not presented in the diagram. However, a detailed description was provided in the results section. In figure 2, the numbers outside the parentheses represent the strength of the relationships between variables. The numbers inside the parentheses are the total effects, which are the sum of the direct effects and the indirect effects.

3.) Please explain more detailed the “pain path map” shown in Figure 2.

Response: Thanks for your comments. In Figure 2, in the relationship of "HA adaptation level (X) → delta and beta brainwaves in the parietal lobe (M) → pain perception (Y)", the delta and beta brainwaves in the parietal lobe are the mediating variables. The influence of the independent variable does not directly act on the dependent variable but is transmitted through this intermediate link (the mediating variable). The activity changes of the delta and beta brainwaves in the parietal lobe play a mediating role between the HA adaptation level and the high-altitude pain perception. That is, the high-altitude adaptation level first causes the activity changes of the delta and beta in the parietal lobe, and this change further affects the individual's perception of pain.

**Discussion:**

1.) Please be more structured in your discussion and discuss your findings rigorously. Add a separate text section discussing the limitations of your study!

Response: Thanks for your comments. I have conducted a comprehensive reorganization of the discussion part within the article, thereby rendering it more logical and methodically arranged. Indeed, this study has some limitations. Regarding this, I have made the following supplements: (1) The time span of the research may affect the results. Short-term cross-sectional studies may fail to observe long-term changes or effects. Future research should consider longitudinal tracking, which is more conducive to observing how the cerebral cortex and pain perception change as the individual's level of HA adaptation varies. (2) Only 65 participants were included in this study. Such a relatively small sample size may lead to limited generalizability of the results. Future research should enlarge the sample size to ensure that the sample can more comprehensively reflect the true situation of the target population, thereby enhancing the ecological validity of the study. (3) Currently, the understanding of HA pain perception is still not deep and comprehensive enough, and it has not been possible to accurately define the specific type of pain, that is, it is difficult to determine whether it is localized pain in specific parts of the body or diffuse pain that is widely distributed. In view of this, in future research work, more precise, advanced, and high-resolution technical means should be adopted to accurately locate and distinguish the specific body parts involved in pain perception, so as to explore the internal mechanisms and characteristic manifestations of high-altitude pain perception in a more detailed and in-depth manner, thereby filling the gaps and deficiencies in this research field at present and providing a more solid and reliable basis and support for subsequent theoretical development and practical application.

**Reviewer 3**

The provided text delves into the intriguing relationship between altitude acclimatization (AAI) and pain perception, particularly focusing on the role of the parietal cortex. The study highlights a significant correlation between AAI, pain intensity, and the activation of specific frequency bands within the parietal cortex.

A key finding is the negative correlation between parietal δ and β frequencies and pain sensation. This suggests that increased activity in these frequency bands may serve as a protective mechanism against pain. The authors propose that AAI may enhance the brain's ability to process and modulate pain signals, particularly through the parietal cortex.

It's important to note that the study acknowledges the complexity of pain perception and the limitations of relying solely on subjective self-reports. The authors suggest that future research should explore the potential of objective, data-driven methods to quantify pain, such as machine learning approaches.

Overall, this study provides valuable insights into the neurophysiological basis of pain perception at high altitude.

Response: We appreciate your summary of the manuscript and encouraging comment! In the future, I will spare no effort to extensively search for and deeply study the cutting-edge and authoritative academic materials at home and abroad. I will focus on the classic and time-honored theoretical masterpieces as well as the newly published advanced research reports in related fields, learn more advanced technologies, and deeply explore various influencing factors and the complex and delicate internal operating mechanisms hidden behind the pain perception phenomenon of high-altitude residents. I sincerely hope that I could get more help and support from you!
