# Supplementary material for: Parietal-specific activation reveals pain from inadequate levels of altitude acclimatization/ adaptation
Source: PLoS One. 2025 Jul 15;20(7):e0326385. doi: 10.1371/journal.pone.0326385 (PMC12262904; doi:10.1371/journal.pone.0326385)
Supplement: Attachment — Submitted filename: Response_to_Reviewers_auresp_2.doc [file pone.0326385.s005.doc]

**Title:** Parietal-specific activation reveals pain from inadequate levels of altitude acclimatization / adaptation

**Corresponding author:** Hao Li

**Date of review**: 05/03/2025

**General comment**

Thank you for the thorough revision of your manuscript. I appreciate the effort you put into addressing my comments/questions as well as those from the other reviewers. Your detailed and comprehensive responses to all of my questions are commendable, and the changes you made have significantly improved the quality and clarity of the manuscript. I have only a few remaining minor remarks, mostly related to wording suggestions or slight reformulations that could further enhance the readability of the text.

**Page 4**

Replace:

“All participants given written informed consent and paid for their participations.”

By:

All participants gave their written informed consent and were compensated for their participation.

**Pages 6-7**

I would recommend replacing:

“2.7 statistical analyses

Data analysis was conducted using SPSS 21.0. Based on the core issue of this study which focuses on exploring the intrinsic link between the HA adaptation level and pain, the AAI index was selected as the key physiological indicator to reflect the HA acclimatization / adaptation level, and the score of the pain frequency, intensity, and burden scale (P - FIBS) was used to quantify the pain level of individuals. The independent-samples *t*-test was adopted to evaluate the pain levels of individuals under different acclimatization / adaptation levels. Pearson correlation analysis was adopted to measure the correlation degree between the AAI and the P - FIBS score through the correlation coefficient (r). *P* <.05 indicates that the results are significant and possess statistical significance. Based on the theoretical frameworks of previous studies and this current study, in view of the potential crucial role of the electroencephalogram activities in different frequency bands of the parietal lobe in the conduction pathway between the AAI index and pain perception, it was selected as the mediating variable. The regression-based mediation effect analysis method was applied to construct the following models: Model 1, with the AAI index as the independent variable and pain as the dependent variable, to test the total effect; Model 2, with the AAI index as the independent variable and the electroencephalogram activities in different frequency bands of the parietal lobe as the dependent variable, to examine the influence of the independent variable on the mediating variable; Model 3, incorporating both the AAI index and the electroencephalogram activities in different frequency bands of the parietal lobe simultaneously, with pain as the dependent variable, to analyze the direct effect of the independent variable on the dependent variable and the indirect effect of the mediating variable after the introduction of the mediating variable. To obtain more robust estimates of the mediating effect and confidence intervals, the Bootstrap method was adopted, with the number of resampling times set to 5000. Through this extensive resampling process, an empirical sampling distribution of the mediating effect was constructed, and then its 95% confidence interval was determined. If this interval does not include 0, it indicates that the mediating effect is significantly present.”

By:

2.7 Statistical analyses

Data analysis was performed using SPSS version 21.0. Given the central aim of the study—to

investigate the intrinsic relationship between HA adaptation and pain—the AAI index was chosen as the main physiological marker to reflect the level of HA acclimatization/adaptation. Pain was quantified using the P-FIBS. An independent-samples t-test was conducted to compare pain levels across different levels of HA adaptation. Pearson correlation analysis was used to assess the strength of association between the AAI index and P-FIBS scores, with significance determined at p < .05. Drawing on prior theoretical frameworks and the present study’s rationale, EEG activity in various parietal lobe frequency bands—due to its potential key role in the neural pathway between HA adaptation and pain perception—was introduced as a mediating variable. A regression-based mediation analysis was conducted using the following models:

Model 1: AAI as the independent variable and pain as the dependent variable (total effect);

Model 2: AAI as the independent variable and EEG activity as the dependent variable (effect on the mediator);

Model 3: both AAI and EEG activity as predictors, with pain as the dependent variable, to estimate the direct and indirect effects. To improve the robustness of the mediation estimates, the Bootstrap method was applied with 5000 resamples. This approach yielded an empirical distribution of the mediation effect and its 95% confidence interval. A confidence interval that does not include zero indicates a statistically significant mediation effect.

**Page 7**

I would recommend replacing:

"Secondly, a significant negative correlation was indicated between pain and δ frequencies (r = -0.31, p = 0.01). Lastly, AAI was significantly positively correlated with parietal lobe activity. Among them, it showed significant positive correlations with the δ frequency (r = 0.28, p = 0.02) as well as with the β frequency (r = 0.26, p = 0.04). No other significant correlations were observed aside from those related to EEG frequencies (Table 3)."

By :

Second, a significant negative correlation was observed between pain and δ frequency activity (r = - 0.31, p = 0.01), suggesting that greater δ activity was linked to reduced pain. Finally, AAI was positively correlated with parietal EEG activity, showing significant associations with both δ frequency (r = 0.28, p = 0.02) and β frequency (r = 0.26, p = 0.04). No other significant correlations were found outside of those involving EEG frequency bands (Table 3).

**Page 8**

I would recommend replacing:

"Among them, when δ was the mediating variable, the total effect was -1.20 (p = 0.04, 95%CI [-2.35, - 0.04], not including 0). The direct effect was -0.85 (p = 0.15, 95%CI [-2.03, 0.32], including 0), and the indirect effect of δ frequency was -0.35 (95%CI [-1.21, -0.01], not including 0), accounting for 29% of the total effect (see Table 5). Similarly, when β served as the mediating variable, the total effect was - 1.20 (p = 0.04, 95%CI [-2.35, -0.04], not including 0). The direct effect was -1.51 (p = 0.01, 95%CI [-2.68, -0.34], not including 0), and the indirect effect of β frequency was 0.31 (95%CI [0.00, 1.16], not including 0), accounting for 21% of the total effect (see Table 7). Therefore, the δ and β frequencies in the parietal lobe were identified as the crucial mediating variables between AAI and pain."

By

Among them, when δ frequency was tested as a mediating variable, the total effect of AAI on pain was significant (total effect = -1.20, *p* = 0.04, 95% CI [-2.35, -0.04], excluding 0). The direct effect was not statistically significant (direct effect = -0.85, *p* = 0.15, 95% CI [-2.03, 0.32], including 0), whereas the indirect effect through δ frequency was significant (indirect effect = -0.35, 95% CI [-1.21, -0.01], excluding 0), accounting for 29% of the total effect (see Table 5). Similarly, when β frequency was examined as the mediator, the total effect remained significant (total effect = -1.20, *p* = 0.04, 95% CI [- 2.35, -0.04], excluding 0). In this case, the direct effect was also significant (direct effect = -1.51, *p* = 0.01, 95% CI [-2.68, -0.34], excluding 0), and the indirect effect via β frequency was positive and significant (indirect effect = 0.31, 95% CI [0.00, 1.16], excluding 0), representing 21% of the total effect

(see Table 7). These findings indicate that both δ and β parietal EEG frequencies significantly mediated the relationship between AAI and pain.

**Pages 10-11**

I would recommend replacing:

"In this research endeavor, we have exerted ourselves to optimize our experiments. Nevertheless, akin to all scientific exploration, our work is not devoid of limitations. (1) The time span of the research may affect the results. Short-term cross-sectional studies may fail to observe long-term changes or effects. Future research should consider longitudinal tracking, which is more conducive to observing how the cerebral cortex and pain perception change as the individual's level of HA acclimatization / adaptation varies. (2) Only 65 participants were included in this study. Such a relatively small sample size may lead to limited generalizability of the results. Future research should enlarge the sample size to ensure that the sample can more comprehensively reflect the true situation of the target population, thereby enhancing the ecological validity of the study. (3) Currently, the understanding of HA pain perception is still not deep and comprehensive enough, and it has not been possible to accurately define the

specific type of pain, that is, it is difficult to determine whether it is localized pain in specific parts of the body or diffuse pain that is widely distributed. In view ofthis, in future research work, more precise, advanced, and high-resolution technical means should be adopted to accurately locate and distinguish the specific body parts involved in pain perception, so as to explore the internal mechanisms and characteristic manifestations of HA pain perception in a more detailed and indepth manner, thereby filling the gaps and deficiencies in this research field at present and providing a more solid and reliable basis and support for subsequent theoretical development and practical application.”

**By :**

In this research, we have made every effort to optimize our experimental procedures. However, as with any scientific investigation, our study presents certain limitations. (1) The cross-sectional design may limit the ability to capture long-term changes or effects. Short-term observations may overlook the dynamic evolution of cerebral activity and pain perception as individuals adapt to HA conditions. Future studies would benefit from adopting a longitudinal approach to better track these changes over time. (2) The sample size was relatively limited, with only 65 participants. This small cohort may constrain the generalizability of the findings. Expanding the sample in future studies would enhance the representativeness of the results and improve the ecological validity of the research. (3) Current understanding of pain perception at high altitude remains incomplete. Specifically, it remains unclear whether the pain reported is localized to specific body regions or more diffuse in nature. To address this, future research should incorporate more precise and high-resolution methodologies to accurately identify and differentiate the bodily sources of pain. Such approaches would allow for a deeper investigation of the underlying mechanisms and distinctive features of HA-related pain, helping to fill existing knowledge gaps and providing a stronger foundation for both theoretical advancement and practical applications.

**Response: I am profoundly grateful for the constructive suggestions you provided. All specified sections requiring revision have been systematically replaced in accordance with your recommendations. Through your expert guidance, these modifications have indeed optimized the textual clarity and terminological precision. For future scholarly compositions, I shall rigorously implement such professional discourse patterns to align with academic writing standards."**

**Figures**

You should add a title to both figures.

**Response: I sincerely appreciate your notification. Due to an oversight on my part, the title of both figures were inadvertently omitted. These have now been supplemented in their designated positions within the manuscript.**

***Figure 2***

I would recommend clarifying what the numbers on the figure correspond to. For example : Path diagram showing path coefficients. Total effects are in parentheses.

**Response: Thank you very much for your recommendation. I have made modifications to the captions in the manuscript, and have indicated the meaning of each path. Specifically, a*b = Indirect Effect; c’=Direct Effect; c=Total Effect. (A) AAI, δ frequency and pain path map. (B) AAI, β frequency and pain path map.**
